# Allosteric-activation mechanism of BK channel gating ring triggered by calcium ions

**DOI:** 10.1371/journal.pone.0182067

**Published:** 2017-09-27

**Authors:** Ronghua Guan, Hui Zhou, Junwei Li, Shaoying Xiao, Chunli Pang, Yafei Chen, Xiangrong Du, Shaoxi Ke, Qiongyao Tang, Jiguo Su, Yong Zhan, Hailong An

**Affiliations:** 1 School of Mathematical and Physical Science, North China Electric Power University, Baoding, China; 2 Key Laboratory of Molecular Biophysics, Hebei Province, Institute of Biophysics, School of Sciences, Hebei University of Technology, Tianjin, China; 3 School of Architecture & Art Design of Hebei University of Technology, Tianjin, China; 4 Jiangsu Province Key Laboratory of Anesthesiology, Xuzhou Medical University, Xuzhou, Jiangsu Province, China; 5 College of Science, Yanshan University, Qinhuangdao, China; Zhejiang University Life Science Institute, CHINA

## Abstract

Calcium ions bind at the gating ring which triggers the gating of BK channels. However, the allosteric mechanism by which Ca^2+^ regulates the gating of BK channels remains obscure. Here, we applied Molecular Dynamics (MD) and Targeted MD to the integrated gating ring of BK channels, and achieved the transition from the closed state to a half-open state. Our date show that the distances of the diagonal subunits increase from 41.0 Å at closed state to 45.7Å or 46.4 Å at a half-open state. It is the rotatory motion and flower-opening like motion of the gating rings which are thought to pull the bundle crossing gate to open ultimately. Compared with the ‘Ca^2+^ bowl’ at RCK2, the RCK1 Ca^2+^ sites make more contribution to opening the channel. The allosteric motions of the gating ring are regulated by three group of interactions. The first weakened group is thought to stabilize the close state; the second strengthened group is thought to stabilize the open state; the third group thought to lead AC region forming the CTD pore to coordinated motion, which exquisitely regulates the conformational changes during the opening of BK channels by Ca^2+^.

## Introduction

Large conductance, Ca^2+^-activated potassium (BK) channels are one type of calcium-activated potassium channels. BK channels are known as Big K^+^ channels, which is due to having a large single-channel conductance of ~100–300 pS.[1] BK channels are widely expressed throughout the animal kingdom, which play important roles in many physiological processes, such as ansmitter release[2], secretion of endocrine[3], and regulation of vascular[4]. Loss-function of BK channels could lead to epilepsy[5], hypertension[6], asthma[7], tumor progression[8], obesity[9].

Similar to voltage-gated K^+^ channels, BK channels are a tetramer of the pore-forming subunits, which possess a voltage-sensor domain (S1-S4) that senses membrane potential changes, a pore-gate domain (S5-S6) that opens and closes to control ion selectivity and K^+^ permeation, and a large cytosolic tail domain (CTD) that forms a gating-ring serving as the primary ligand sensor, which is sensitive to intracellular chemical ligands such as Ca^2+^[10–12] and others[13–15]. The main structural components of gating ring are two regulators of K^+^ conductance (RCK) domains (RCK1 and RCK2) that are connected by a∼100-amino acid linker[16]. Each RCK domain can be further divided into three subdomains: Rossmann-fold subdomain (βA–βF), which contains a AC region (βA, αA, αB and βB) which forms the CTD pore; intermediate helix-crossover (αF-turn-αG); and C-terminal subdomain (αH–C-terminus).[17]

Electrophysiological and mutagenesis experiments have identified two Ca^2+^ high affinity binding sites for each subunits: one is located in RCK1 domain including the residues of D367, R514 and E535[18], which are called ‘RCK1 Sites’, and the other in the C-terminus of RCK2 domain, containing a string of Asp residues known as the ‘Ca^2+^ bowl’.[19] BK channels gain Ca^2+^ sensitivity by their association with Ca^2+^-binding calmodulin proteins.[20] Ca^2+^ binding stabilizes the conducting state of the channel, which shows that Ca^2+^ induces conformational rearrangements of the gating ring and open the transmembrane and CTD pores.

Recently, three crystal structures of eukaryotic CTD of BK channels (PDB ID: 3MT5, 3NAF and 3U6N), including both RCK1 and RCK2, respectively.[17, 21, 22] The x-ray structure of the human BK Ca^2+^gating (PDB: 3MT5) was firstly solved and deduced its tetrameric assembly by structure of a Na^+^-activated homolog.[21] The crystal structure of the entire cytoplasmic region of the human BK channel in a Ca^2+^ free state (PDB:3NAF) reveals four intracellular subunits and the linker connecting S6 and gating ring, which can generate a structural model for full BK channel.[17] The crystal structure of zebrafish BK channel in Ca^2+^-bound state with eight subunits(PDB:3U6N), shows that one layer of gating ring opens upon binding Ca^2+^. Those crystal structures present molecular bases for homolog modeling and conformational transition pathway with Ca^2+^ opening BK channels.

With the collective efforts of the BK channels field, the understanding of molecular mechanisms of BK channel function has been greatly advanced over the past three decades,[23] but it is still not clear that the molecular mechanism of intracellular Ca^2+^-induced conformational changes of BK channel gating ring. So, we should first address the follow questions: 1) Which is more important to widen the gating ring aperture, RCK1 or RCK2? 2) How does the interaction deliver during the BK channel gating?

Here, the authors combined Molecular Dynamics (MD) with Targeted MD on the gating ring of BK channels, and achieved the transition from the closed state to a half-open state. Our data indicate that the RCK1 Ca^2+^ sites make more contribution to opening the channel than the RCK2 domains do. We identified a series of interaction networks, which regulates the conformational changes during the opening of BK channels by Ca^2+^.

## Materials and methods

### Homology modeling

The structures of gating ring were taken from homology models of the CTD of the BK channels based on the closed and open state models of crystal structure of gating ring (PDB ID:3NAF and 3U6N).[17, 22] Crystal structures were retrieved from protein data bank (www.rcsb.org). The target sequences were taken from protein data bank (PDB ID: 3U6N). Homology models of the BK channel gating ring were all based on chain A of the template structures using SWISS-MODEL server.[24–26] These models were evaluated with GMQE.[27, 28] GMQE (Global Model Quality Estimation) is a quality estimation which combines properties from the target-template alignment. The resulting GMQE score is expressed as a number between 0 and 1, reflecting the expected accuracy of a model built with that alignment and template. Higher numbers indicate higher reliability. The members of the BK channel family show high degree of sequence similarity. Due to high sequence identity (about 96.89% and 95.68%), the GMQE score is 0.90, and 0.71 respectively. [29] Compared to its templates, the mainchain geometry in experimental models had no change. We applied transformation matrix from the PDB file (3NAF and 3U6N) to generate the complete tetramer of closed and open states of gating ring, respectively.[17, 22]

### Conventional molecular dynamics

The Molecular dynamics (MD) simulations with explicit solvent and ions were carried out on two separate systems (closed and open states) of the gating ring of BK channel in ~150 mM KCl [30]. The K^+^ and Cl^-^ were positioned randomly in a rectangular box of water with the size of 183 ×183 × 86 Å^3^ (closed state), and 162 × 116× 175 Å^3^ (open state), respectively. The water potential TIP3P was used[31].

The minimization and molecular dynamics simulations were carried out using the NAMD2 program(http://www.ks.uiuc.edu/Research/namd/)[32] and the CHARMM 27 force filed[33]. During the production run, a 2.0 kcal/mol harmonic restraint on the Cα atom of gating ring was maintained for 5 ns. Then, letting the system relax freely for last over 10–20 ns until reaching equilibrium. Langevin dynamics and the Langevin piston were used to maintain the temperature at 310 K and a pressure control, respectively. The van der Waals interactions were modeled using Lennard-Jones. Short-range non-bonded interactions were truncated at 12 Å. Long-range electrostatics was calculated using the particle mesh Ewald (PME) algorithm with grid spacing 1Å.[34] The calculations were performed on every time step, which was 2 fs. Simulation analysis and structural diagrams were used with VMD (Visual Molecular Dynamics).[35]

### Targeted Molecular Dynamics

Targeted Molecular Dynamics (TMD)[36] has been used in studies of allostery and a variety of transitions in large proteins. In TMD, a subset of atoms (target atoms) is guided toward a target structure by means of steering forces which gradually steers the initial structure toward the target structure and is obtained through the gradient of a potential calculated as a function of RMSD, which are defined as Eq (1):

$$U_{TMD}=k˙\;{\left[RMSD\left(t\right)\text{-}RMSD*\left(t\right)\right]}^{2}/2N$$ (1)

where *k* is the force constant, and N is the number of targeted atoms. The number of atoms used to calculate the RMSD from the target structure was set to be the same as the number of restrained atoms. At each time step, the *RMSD*(*t*) between the current coordinates and the target structure was computed (after first superimposing the target structure and the initial coordinates). RMSD*(*t*) evolves linearly from the initial RMSD at the first TMD step to the final RMSD at the last TMD step. RMSD*(*t*) tends to zero is the criterion to end the TMD.

### Principal component analysis

Principal component analysis (PCA) was carried out using Normal Mode Wizard (NMWiz) (http://prody.csb.pitt.edu/nmwiz/) to a trajectory from TMD simulations[37]. Normal Mode Wizard (NMWiz) is a VMD plugin[38, 39] for depiction, animation, and comparative analysis of normal modes. Normal modes may come from principal component of structural ensembles, essential dynamics analysis of simulation trajectories, or normal mode analysis of protein structures. In addition, NMWiz can be used to depict any vector that describes a molecular motion.

The standardized trajectory data is then utilized to generate a covariance matrix between the C_α_ atoms *i* and *j*, which are defined as which are defined as Eq (2):

$$C_{ij}=<(x_{i}-<x_{i}>)(x_{j}-<x_{j}>)>\left(i,j=1,2,3\ldots 3N\right)$$ (2)

Where *x*_*i*_ and *x*_*j*_ are Cartesian coordinates of the *i* th and *j* th Cα atom. *N* is the number of Cα atoms considered. <*x*_*i*_> and <*x*_*j*_> represent the time average over all the configurations obtained in molecular dynamics simulations.[40]

## Results

### Construction and MD simulations on the BK gating ring

**Fig 1:**
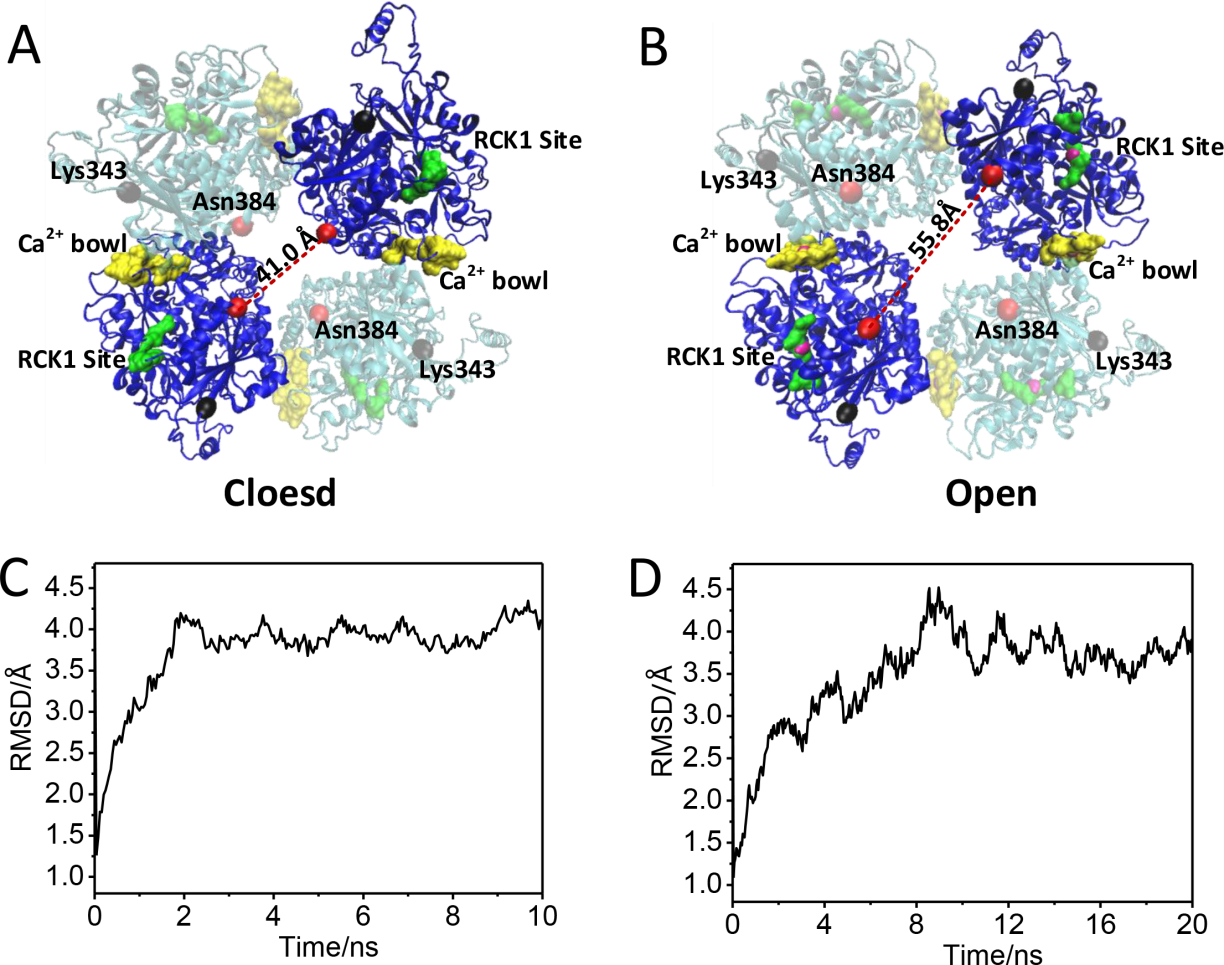
Schematic diagrams of the structures and RMSDs of BK gating ring. (A, B) Schematic diagrams of the structures of BK gating ring in closed and open states, respectively. Cα atoms of Lys343 and Asn384, and Ca^2+^ are represented as black ball, red ball, and magenta ball, respectively. RCK1 site and Ca^2+^ bowl are highlighted as green and yellow, respectively. (C, D) The RMSDs were calculated based on all the Cα atoms of the BK gating ring for close and open states, respectively.

**Fig 2:**
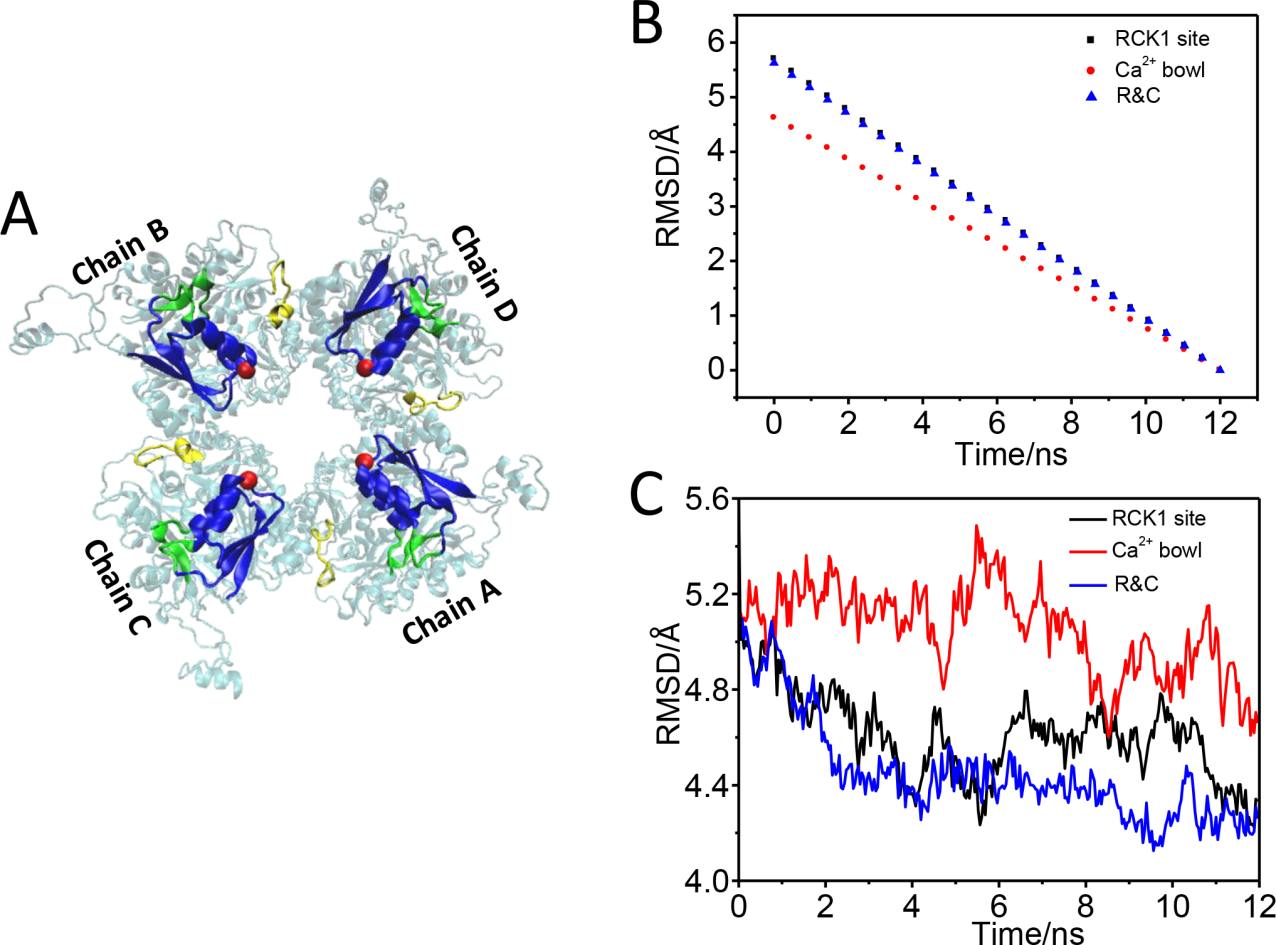
The gating ring achieved a half-open state after targeted MD simulation. (A) The schematic diagram of closed state, the target force is applied to those residues (His365-Asp369, Ser512-Phe516, and Ser533-Tyr537) and (Asn887-Pro899), which are colored in green and yellow, respectively. The main conformational difference happens in the AC region (blue). (B) The evolution of RMSDs from the initial RMSD at the first TMD step to the final RMSD at the last TMD step. (C) RMSDs are calculated based on all the Cα atoms of gating ring along the TMD simulations compared to the target structure.

In present study, we have constructed two 3-dimentional structures of BK gating ring, Ca^2+^-free (closed) and Ca^2+^-bound (open) states (Fig 1A and 1B). During 10–20 ns free MD simulations, these two structures reach their equilibration states because the Cα root-mean-square deviations (RMSDs) values are 4 Å or less (Fig 1C and 1D). The diagonal subunits distances of the closed and open states of the reaching equilibrium were 41.0 Å and 55.8 Å, which were measured at Cα atoms of the N-terminal residues Asn384 (red balls) of the helix αB by virtue if it’s more stabled than the Cα atoms of the N-terminal residues Lys343 (black balls) during MD simulation (Fig 1 and Fig 2A). The RCK1 sites are colored in green, the ‘Ca^2+^-bowl’ sites are colored in yellow.

### The RCK1 domain makes more contribution on opening the BK channel

**Fig 3:**
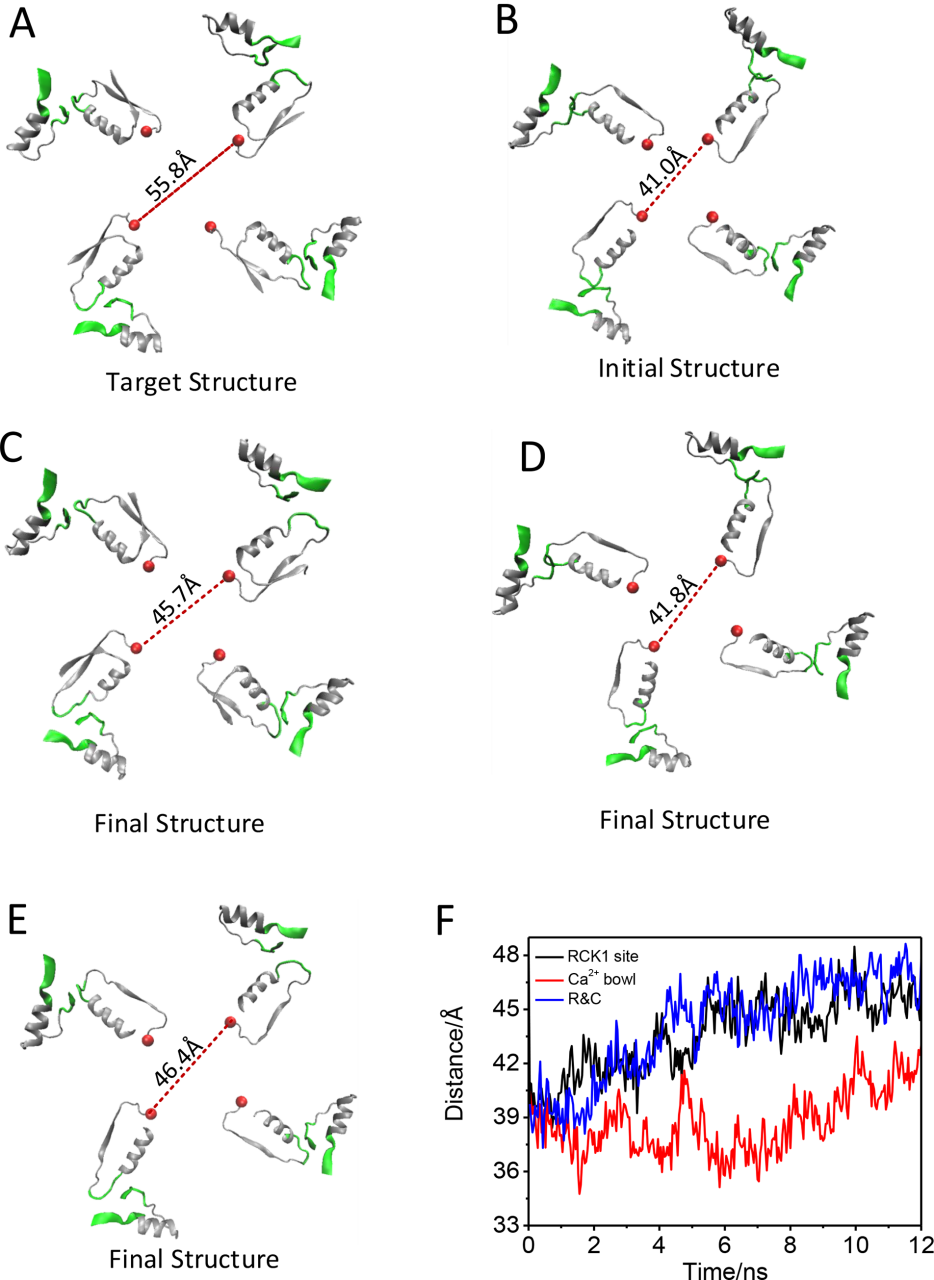
Distances of diagonal subunits of gating ring and RMSD from TMD simulations. (A) The Distance of diagonal subunits of the gating ring in open state (target structure). (B) The Distance of diagonal subunits of the gating ring in closed state (initial structure). (C, D, E) show the distance of diagonal subunits of the gating ring after TMD simulation, whose targeted forces were applied to RCK1 site, Ca^2+^ bowl, RCK1 site & Ca^2+^ bowl (R & C), respectively. (F) Distance of diagonal subunits of the gating ring measured at Cα atoms of residues Asn384 after TMD simulations on binding sites (black), Ca^2+^ bowl (red) and R &C (blue), respectively.

To identify which of the Ca^2+^-binding regions contributes more to opening the BK channel, we carried out three TMD simulations on the RCK1 sites (green), ‘Ca^2+^-bowl’ sites (yellow) and both the two regions, respectively (Fig 2A). The corresponding Ca^2+^-binding regions in BK channel at open state was set as a target structure. During the TMD simulations, an external force was applied to the backbone atoms of the RCK1 domain (His365 to Asp369, Ser512 toPhe516, Ser533 to Tyr537) and RCK2 domain (Asn887 to Pro899) with a force constant of 500 kcal/mol/Å^2^. The Cα RMSD was decreased monotonically from the initial RMSD to near zero Å along the TMD trajectory (Fig 2B), which identified that the three TMD simulations had finished. During this process, the Cα RMSD of BK gating ring between the closed structure in the simulation and the open structure shows that closed gating ring from the TMD on Ca^2+^-binding sites (RCK1 site-black line) is similar to structure from the TMD on Ca^2+^-binding regions (R&C-blue line), whose two structures are closer to the open gating ring than the structure from the TMD on Ca^2+^-bowl sites (Ca^2+^ bowl-red line) (Fig 2C).Further analysis, the distance between the diagonal subunits of gating ring is 45.7 Å, 41.8 Å, 46.4 Å at the end of TMD simulation on RCK1 site, Ca^2+^ bowl and R&C, respectively, which suggests that the gating ring achieved a partial opening, or quasi-opening state with the TMD simulation on the RCK1 site and R&C (Fig 3A–3E). The open processes are consistent with the movement of RMSD along the TMD simulations (Fig 3F). These results suggest that the Ca^2+^-binding sites in RCK1 contribute more than binding to the ‘Ca^2+^ bowl’ sites to opening the BK channel (Figs 2 and 3).

### There are two motion models during the gating of BK channel

**Fig 4:**
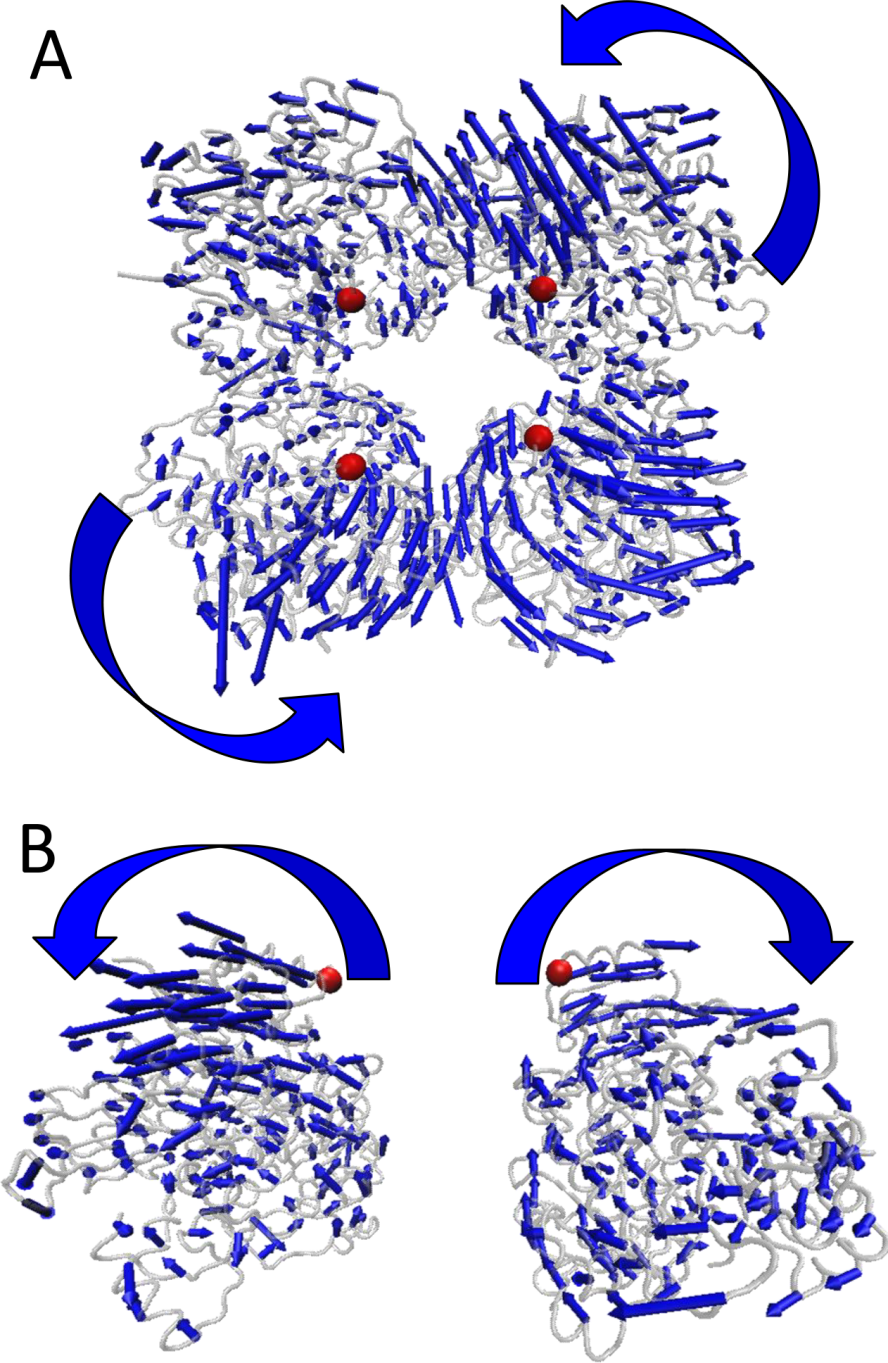
The dynamic motion during gating ring opening from the TMD simulation on RCK1 sites. (A) and (B) show the first principal component of the two motion tendency of gating ring during the TMD simulation on the RCK1 sites.

To explore the dynamics behavior of gating ring based on the TMD simulation trajectory, essential dynamics analysis was conducted. The first principal component of motion tendency of gating ring based on the TMD simulation trajectory is shown in Fig 4. It can be illustrated that the gating ring experiences an anticlockwise rotational motion around the gating ring axis and a flower-opening like motion which push the channel to open state. These two dynamic motions of gating ring can be seen obviously from the calculation results of essential dynamics analysis using the animation function of VMD 1.9.2 plugin.[39]

### Interaction-networks exquisitely regulate the gating of BK channels

**Fig 5:**
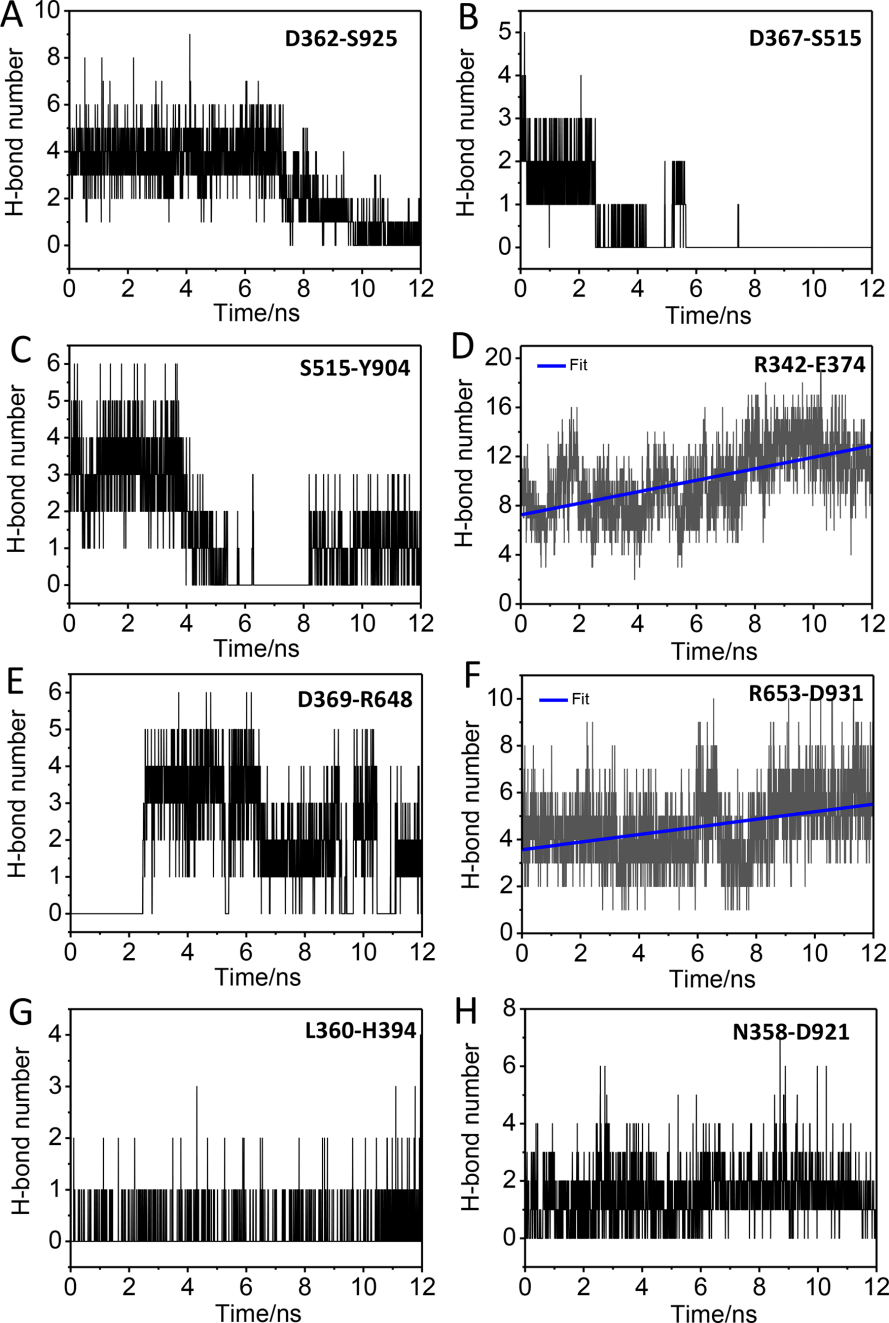
Time course of interactions that regulate the gating of BK channels. (A), (B), and (C) show the time course of the interactions between D362 and S925, D367 and S515, S515 and Y904. (D), (E) and (F) show the time course of the interactions between R342 and E374, D369 and R648, R653 and D931. (G, H) The time course of the interactions between L360 and H394, N358 and D921.

**Fig 6:**
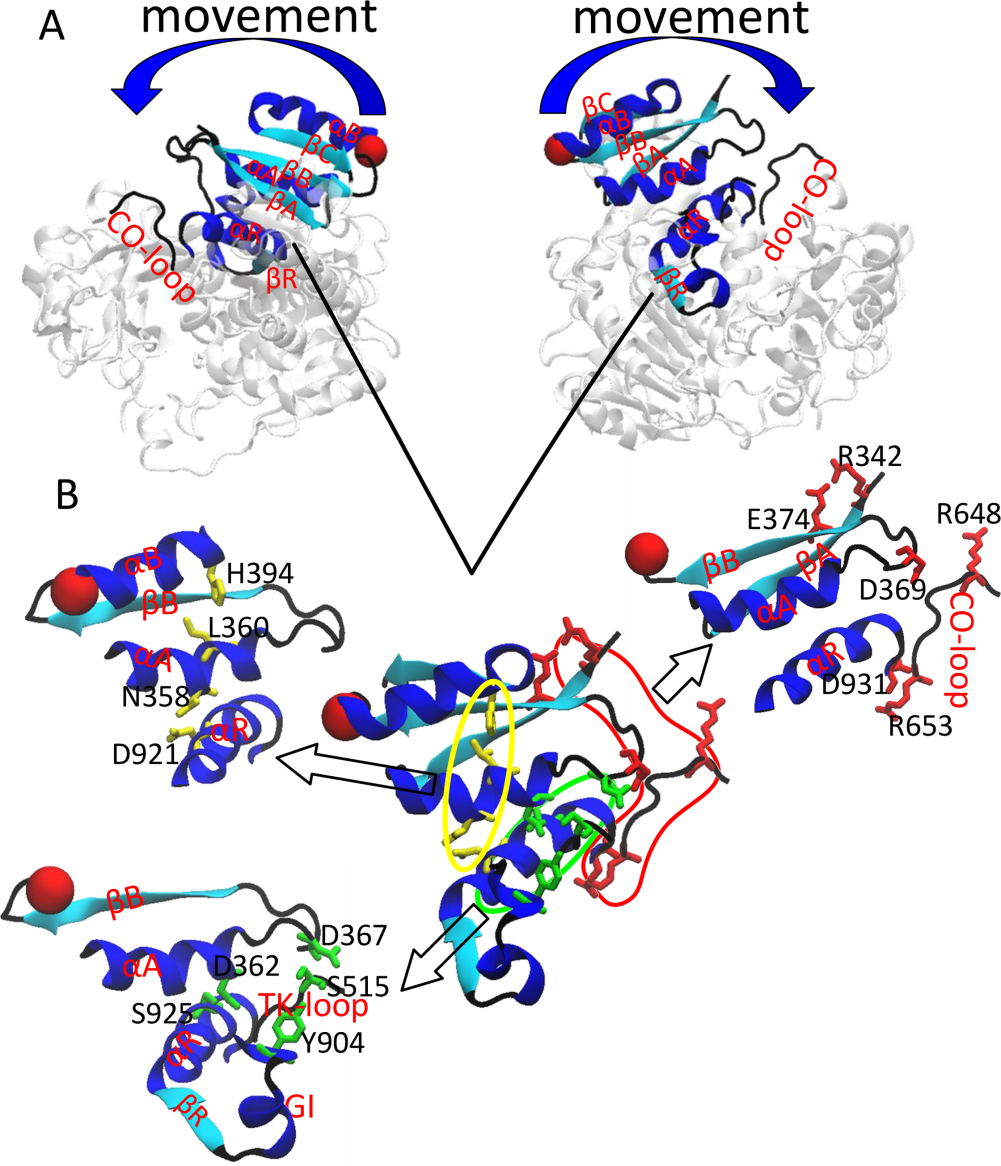
The transmission pathway of interactions during BK gating ring opening. (A) A side view of BK gating ring with top surface facing the membrane shows where the transmission pathway of interactions. (B) An enlarged view of the transmission pathway of interactions. The green ellipse represents the weakened interactions. The red region represents the strengthened interactions, which is like a hooked arm. The yellow ellipse represents the interactions, which lead the entire AC region to coordinated motion.

To identify the transmission of interaction that brought about the dynamic motion of gating ring, inner weak interactions analysis was conducted. We identified three interaction-networks. In the gating ring opening process, one of the interaction-networks is broken, which consist of three pairs of interactions, between αA and αR (D362-S925), αA-βB loop and TK-loop (T:turn) (D367-S515), TK-loop and GI (G:3/10-helix) (S515-Y904), respectively (Figs 5A–5C and 6); the other interaction-network also contains three pairs of interactions between βA and βB (R342-E374), αA-βB loop and CO-loop (C:coil) (D369-R648), CO-loop and αR (R653-D931), which are strengthened, respectively (Figs 5D–5F and 6); the third consists of the interactions between αA and αB (L360-H394), αA and αR (N358-D821) (Figs 5H–5G and 6). The network of interactions also exist in the ACregion which is formed by αA, αB, βA and βB. Our data show that there are two parts that facilitates the movement of AC region, one is the weakened interactions (green ellipse) that liberate the AC region (Fig 6B), and the other is the interactions within AC region that make the entire AC region coordination (yellow ellipse) (Fig 6B). The three pairs of strengthened interactions is like a hooked arm (red region) to make the AC region movement of the open state, (The interactions between D369 and R648, between R653 and D931, between R342 and E374 as an elbow, a shoulder and a hand, respectively.) (Fig 6B).

## Discussion

Ca^2+^-induced gating of BK channel is an intrinsically dynamic process. However, since allosteric conformational changes take place on the microsecond time scale, it is not possible to capture the transfer of a closed state to an open state even through MD simulations. To accomplish the transition from the closed to the open state of BK channels, we performed Targeted MD simulations, which was developed by Schlitter et al [36] and has been used in studies of allostery and a variety of transitions in large proteins [41]. Limited by the methods, there is no Ca^2+^ in our Targeted MD simulations. During the simulations, we applied force on the Ca^2+^ binding residues and hoped that the applied forces, in somehow, mimic the channel-Ca^2+^ interactions.

In TMD simulations, a subset of target atoms (RCK1 site, Ca^2+^ bowl or R&C) is guided towards a target structure by means of steering forces, which show that Ca^2+^-binding site in RCK1 is more important than binding to the Ca^2+^ bowl to activate the BK channel gating ring (Figs 2 and 3). Our simulation data are consistent with the experimental results.[13, 42, 43] We next analyzed the global motion of the BK channel gating ring, which exhibits two motions: the flower-opening like motion and the rotation motion (Fig 4). The expansion motion indirectly induced the AC region widen. In a full-length BK channel, the AC region, at the N-terminus of RCK1, is connected to C-terminus of the transmembrane inner helix (S6), which forms the pore’s gate via the S6–RCK1 linker and could therefore be a point of convergence for the conformational changes evoked by Ca^2+^ binding to either RCK1.[43] The rotation of the gating ring may pull the conformational changes of the S6–RCK1 linker to open the activation gates of BK channel, which is similar to PIP_2_ opening Kir channels.[23, 41]

The two gating ring structures, Ca^2+^-bound and Ca^2+^-free states, differ in position that the RCK1 layer in the Ca^2+^-bound gating ring is expanded from a diameter of 81 to 93 Å, measured at the position of Lys343s.[22] In the full BK channel, the Lys343s locate in S6–RCK1 linker that connects the transmembrane domain to cytosolic tail domain (gating ring)[22, 44]. The S6–RCK1 linker may undergo conformational change during opening of the activation gates. Our models only consist of gating ring, absence of the entire transmembrane spanning domains, the fluctuations of Lys343s may be wider during simulations. So the diagonal distance of Lys343s cannot show the accurate distance of the pore gate of gating ring. We chose the Asn384s (red ball) as the position to measure distance of gating ring which expanded from 41.0 to 55.8 Å from closed state to open state (Fig 3A and 3B).

The gating ring opening to the target structure during TMD simulations with RCK1 sites, we identified that three interaction-networks played a critical role in the Ca^2+^-induced gating of BK channels (Fig 5). The weakened interactions decrease the correlation between the AC region and around the RCK1 region (green ellipse) (Fig 6B), which facilitate the AC region motion, the interactions within AC region help to keep stability and coordination of AC region (yellow ellipse) (Fig 6B), the strengthened interactions pull AC region to expand, which is like a hooked arm (red region) (Fig 6B). These interaction-networks make the AC region to movement of the open state. By analyzing the relationship between motions of gating ring and interactions, we identified that the transmission pathway of interaction during BK gating ring opening. The small conformational changes in RCK1 site (Ca^2+^ binding RCK1 site) induced the large conformational changes of the gating ring.
